# Supply chain logistics – the role of the Golgi complex in extracellular matrix production and maintenance

**DOI:** 10.1242/jcs.258879

**Published:** 2022-01-13

**Authors:** John Hellicar, Nicola L. Stevenson, David J. Stephens, Martin Lowe

**Affiliations:** 1 School of Biological Sciences, Faculty of Biology, Medicine and Health, University of Manchester, The Michael Smith Building, Oxford Road, Manchester, M13 9PT, UK; 2 Institute of Molecular and Cell Biology (IMCB), Agency for Science, Technology and Research (A*STAR), 61 Biopolis Drive, Proteos, Singapore, 138673; 3 Cell Biology Laboratories, School of Biochemistry, Faculty of Life Sciences, University Walk, University of Bristol, Bristol, BS8 1TD, UK

**Keywords:** Golgi complex, Collagen, Extracellular matrix, Glycosylation, Proteoglycans, Secretory pathway

## Abstract

The biomechanical and biochemical properties of connective tissues are determined by the composition and quality of their extracellular matrix. This, in turn, is highly dependent on the function and organisation of the secretory pathway. The Golgi complex plays a vital role in directing matrix output by co-ordinating the post-translational modification and proteolytic processing of matrix components prior to their secretion. These modifications have broad impacts on the secretion and subsequent assembly of matrix components, as well as their function in the extracellular environment. In this Review, we highlight the role of the Golgi in the formation of an adaptable, healthy matrix, with a focus on proteoglycan and procollagen secretion as example cargoes. We then discuss the impact of Golgi dysfunction on connective tissue in the context of human disease and ageing.

## Abstract (precis)

**Summary:** This Review describes current knowledge on the major role of the Golgi apparatus, the central organelle of the secretory pathway, in the production and maintenance of the extracellular matrix.

## Introduction

The extracellular matrix (ECM) is a complex, highly ordered network of proteins and attached carbohydrates, which provides structural support to tissues while dynamically influencing cellular processes, such as proliferation, differentiation and migration. The ‘matrisome’ itself consists of ∼300 distinct proteins (Shao et al., 2020), the most abundant of which are the collagens. These form the basic support structure of tissues and thus determine their biomechanical properties, for example building long elastic fibres in tendons (Kannus, 2000) or a transparent layered lattice in the cornea (Meek, 2009). Collagens also provide a surface for integrin-mediated cell adhesion and can bind and store growth factors and other signalling molecules. Other major fibrous proteins contributing to ECM structure include fibronectin, elastin, laminins and tenascin (Frantz et al., 2010; Karamanos et al., 2021). The ECM also contains an abundance of proteoglycans, all composed of a core protein modified with one of six types of sulfated glycosaminoglycan (GAG) chains (Iozzo and Schaefer, 2015; Schwartz, 2000). These large linear polysaccharides have negatively charged carboxyl and sulfate groups, which are important for attracting water into tissues. They also regulate a wide range of biological functions as reviewed previously (Raman et al., 2005).

Matrix components are synthesised within the secretory pathway prior to exocytosis and assembly in the ECM. However, their size and extensive glycosylation pose many challenges for the cellular machinery. For example, fibrillar procollagen molecules can measure more than 300 nm in length (Bruns et al., 1979), far exceeding the diameter of traditional transport carriers, whereas the proteoglycan aggrecan accumulates ∼100 GAG chains (Kiani et al., 2002), increasing in molecular mass 10-fold during transit. Furthermore, many ECM proteins, including procollagen, are synthesised as pro-proteins that need processing, and often carry post-translational modifications that must be tailored to suit specific tissues, developmental stage or injury (Frantz et al., 2010; Walma and Yamada, 2020).

**Fig. 1.**
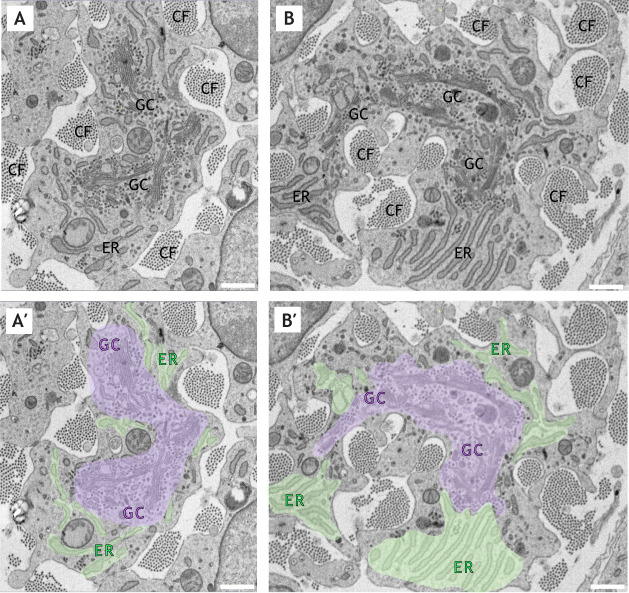
**Electron microscopy of the Golgi in mouse tendon fibroblast cells.** (A,B) Images of two separate fibroblasts present in mouse tendon at embryonic day 17, imaged using high-resolution scanning electron microscopy. The Golgi complex (GC), endoplasmic reticulum (ER) and bundles of collagen fibres (CF) are indicated. A′ and B′ show colour-coded representations of A and B, respectively, with the Golgi highlighted in magenta and the ER in green. Note the prominent Golgi in the tendon fibroblasts, where it occupies a significant proportion of the cell area in the sections shown. The images were taken from 3D block face scanning electron microscopy series that are shown in Movies 1 and 2, respectively. Images courtesy of Professor Karl Kadler and Dr Yinhui Lu, Wellcome Centre for Cell-Matrix Research, University of Manchester, UK. Scale bars: 1 µm.

The majority of the challenges of post-translational modification and processing of ECM proteins are met by the Golgi complex. Indeed, cells that produce large quantities of ECM, such as osteoblasts, chondrocytes and fibroblasts, have a prominent Golgi (Capulli et al., 2014; Florencio-Silva et al., 2015; Hascall, 1980) (Fig. 1; Movies 1 and 2). Although the capture of large ECM cargoes in endoplasmic reticulum (ER)-derived vesicles has previously attracted much attention (McCaughey and Stephens, 2019), the interplay between cargo modification and transit through the Golgi is an equally important topic, with errors in these processes leading to tissue malfunction. This Review will discuss the role of the Golgi as a central control point for ECM synthesis and maintenance, as well as the implications of this in health and disease. We will concentrate on two major classes of ECM proteins as case studies, namely the proteoglycans and fibrillar collagens. The Golgi is also relevant to matrix degradation, but this is not the focus here.

## The central role of the Golgi complex

**Fig. 2.**
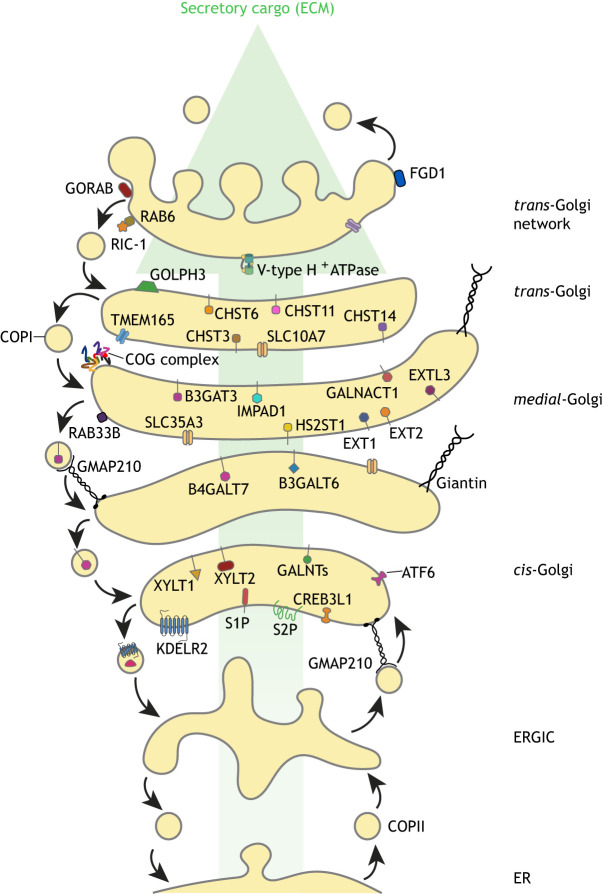
**The Golgi and Golgi proteins associated with ECM-related human disease.** The Golgi acts as a hub for post-translational modification and cargo sorting within the cell. Cargo arrives at the *cis*-face of the Golgi from the endoplasmic reticulum (ER) via the ER-to-Golgi intermediate compartment (ERGIC). As it transports through the Golgi stacks, the cargo undergoes modification by a range of enzymes, which usually act sequentially. These enzymes are themselves trafficked within the Golgi in retrograde COPI-coated vesicles in order to maintain their retention and Golgi homeostasis. As cargo transits through the Golgi stacks, it reaches the *trans*-Golgi network, where it is sorted for delivery to its post-Golgi destination, which, in the case of ECM components, is the plasma membrane from where it is secreted. Shown in this diagram are some key Golgi proteins, including enzymes (depicted as lollipops), ion pumps and transporters, and trafficking proteins, that are known to be mutated in human diseases in which a matrix defect is a major component of the disease phenotype (Table 1).

The Golgi complex acts as a hub for post-translational modification and sorting of cargo within the secretory pathway (Nakamura et al., 2012) (Fig. 2). In most vertebrate cells, the Golgi is comprised of stacked cisternae that are laterally connected to form a continuous ribbon located near the cell centre. Newly synthesised secretory cargo exits the ER in COPII-coated carriers and is delivered, via the ER-Golgi intermediate compartment (ERGIC) in vertebrates, to the entry, or *cis*-side, of the Golgi stack (Brandizzi and Barlowe, 2013). Cargo then moves across the Golgi stack, in a *cis* to *trans* direction, accessing the many Golgi-resident enzymes that carry out post-translational modification and processing of the cargo as it does so. Although the mechanism of cargo transit across the Golgi stack is still debated, most evidence, including analysis of procollagen trafficking (Bonfanti et al., 1998), favours the cisternal maturation model whereby cargo is retained within cisternae that progressively migrate and mature as they move across the stack (Glick and Nakano, 2009). Cisternal maturation is driven by the retrograde trafficking of COPI-coated transport vesicles that recycle Golgi-resident enzymes and other membrane constituents from later to earlier cisternae (Beck et al., 2009). COPI vesicles also mediate recycling of escaped ER residents, including chaperones, such as the major procollagen chaperone Hsp47 (also known as SERPINH1), from the *cis*-Golgi and ERGIC back to the ER. The machinery required for COPI trafficking is described in Box 1. The exit station of the Golgi is the *trans*-Golgi network (TGN), where cargo is sorted into carriers for delivery to the appropriate destination (Guo et al., 2014), which for ECM proteins is the cell exterior via exocytosis.

**Box 1. Golgi trafficking machinery**

**Vesicle formation and cargo sorting**

The small GTPase ARF1 (ARF3, ARF4 and ARF5 are also involved in Golgi trafficking) is activated by its guanine nucleotide exchange factors (GEFs) (GBF1 is the major ARF-GEF at the Golgi) (Claude et al., 1999; Sztul et al., 2019). As a result of binding GTP, ARF1 undergoes a conformational change and binds to the Golgi membrane, where it recruits the heptameric coatomer complex that assembles to form the COPI coat (Arakel and Schwappach, 2018). Additional scaffolding factors, such as SCYL1 and GORAB, also contribute to COPI recruitment and/or assembly (Hamlin et al., 2014; Witkos et al., 2019). Cargo is selected for inclusion into COPI vesicles by binding coatomer directly, or through binding to adaptor proteins that bridge the cargo to the coat. Adaptors include the KDEL receptor, which facilitates recycling of escaped resident proteins to the ER (Newstead and Barr, 2020), and GOLPH3, which mediates recycling of numerous Golgi enzymes proteins within the Golgi stack (Welch et al., 2021). Scission of COPI vesicles is poorly understood but appears to be promoted by ARF1 with possible involvement of additional factors such as CtBP/BARS (Arakel and Schwappach, 2018).

**Vesicle tethering and fusion**

COPI vesicles lose their coat following ARF-GAP activity and GTP hydrolysis, and become tethered to their target Golgi membrane. Tethering is mediated by the golgins, which are coiled-coil proteins found on the surface of the Golgi membrane (Gillingham and Munro, 2016; Witkos and Lowe, 2015). Different golgins localise to distinct Golgi regions and capture specific subsets of vesicles to direct transport and maintain cisternal identity (Gillingham and Munro, 2016; Witkos and Lowe, 2015). Additional interactions with RAB GTPases (Homma et al., 2021) and the multi-subunit tethering complex COG, which is a master coordinator of tethering and fusion (Blackburn et al., 2019), facilitate the transition from initial tethering to membrane fusion (Witkos and Lowe, 2017). Fusion represents the final step in transport and is catalysed by the SNARE proteins, which are short coiled-coil proteins that interact in a selective manner to drive membrane fusion (Wang et al., 2017). Post-fusion, RABs are recycled to the cytosol by RAB-GAPs, while SNARE complexes are dissociated by the AAA ATPase NSF and recycled back to the donor compartment to allow further rounds of transport (Homma et al., 2021; Wang et al., 2017).

**Table 1.**
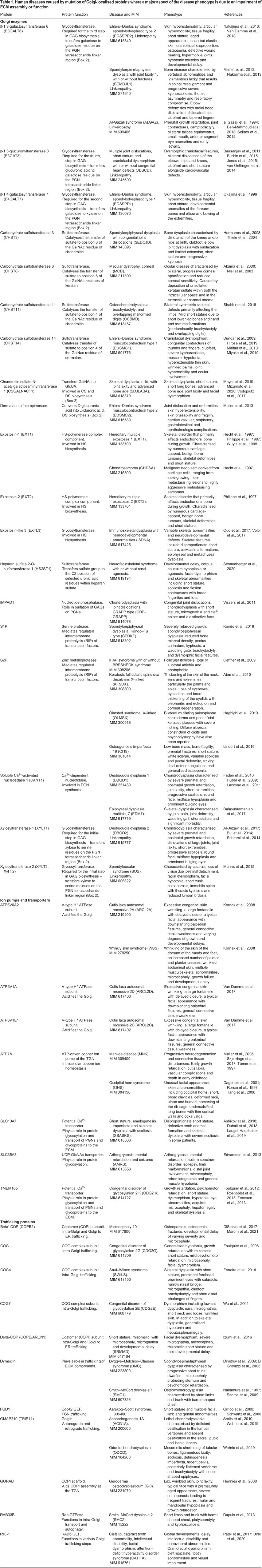
Human diseases caused by mutation of Golgi-localised proteins where a major aspect of the disease phenotype is due to an impairment of ECM assembly or function

### Golgi-based glycosylation

Golgi-resident enzymes mediate a wide range of post-translational modifications, including glycosylation, phosphorylation, sulfation and proteolytic processing (Baeuerle and Huttner, 1987; Moremen et al., 2012; Seidah et al., 2013; Yang et al., 2015). Glycosylation reactions within the Golgi involve the processing of N-linked glycans, whose synthesis is initiated at asparagine residues in the ER, as well as the initiation and processing of O-linked glycans that are attached to serine or threonine residues (Freeze, 2006; Schjoldager et al., 2020). These reactions are mediated by glycosyltransferases, of which there are at least 173 in humans (Schjoldager et al., 2020), and glycosidases, which promote the sequential modification and processing of glycan chains, respectively. Each enzyme has a specific distribution within the Golgi, which suggests a sequential function in glycan chain biosynthesis (Moremen et al., 2012; Schjoldager et al., 2020). Glycosylation is an abundant modification, with 2% of the eukaryotic proteome encoding for glycosylation machinery and ∼85% of secretory proteins receiving this modification (Schjoldager et al., 2020). This includes most ECM proteins, including the very highly glycosylated proteoglycans (PGNs), such as decorin (DCN) and biglycan (BGN) (see below). The combinatorial use of different enzymes allows a large variety of glycan structures to be produced, and the differential expression of enzymes allows the extent and nature of glycosylation to differ depending upon cell type and in response to functional needs (Moremen et al., 2012; Schjoldager et al., 2020).

### Maintaining cisternal identity

Golgi enzymes and other resident proteins are maintained within specific regions of the Golgi despite the extensive flux of cargo through this organelle. This is ensured by COPI-mediated retrieval, which sorts residents into recycling COPI vesicles and delivers them back to the relevant cisternae by binding to golgin tethers (see Box 1). Transmembrane domain (TMD) length also plays a role in Golgi retention, allowing for energetically favourable partitioning of resident proteins into cisternal membranes of a similar bilayer thickness, which is known to increase in a *cis*- to *trans*- direction because of changes in lipid composition (Munro, 1995; Sharpe et al., 2010; Welch and Munro, 2019). It has also been found that Golgi proteins can segregate within cisternae, with Golgi enzymes residing towards the interior of cisternae at steady state, whereas the trafficking machinery is present at the rims (Tie et al., 2018). Bulky cargoes also appear to segregate from smaller soluble cargoes within cisternae (Tie et al., 2018). How intra-cisternal segregation of proteins is maintained is currently unclear, but likely involves lateral protein association, physical occlusion and possibly phase separation (Rebane et al., 2020; Rothman, 2019).

### Golgi-based transcriptional control

In addition to its main role in post-translational modification and sorting of cargo, the Golgi participates in other cellular processes, including transcriptional regulation. An interesting example is that of the membrane-tethered transcriptional factors CREB3L1 and CREB3L2 (cAMP responsive element-binding protein 3 like 1 and 2), which are important for bone and cartilage development (Caengprasath et al., 2021). In response to appropriate osteogenic or chondrogenic stimuli, these factors are cleaved in the Golgi by the zinc metalloproteases S1P and S2P [also known as membrane bound transcription factor peptidases site 1 and 2 (MBTPS1 and MBTPS2)] (Hino et al., 2014; Murakami et al., 2009). This releases N-terminal fragments of CREB3L1 and CREB3L2, which then enter the nucleus and stimulate transcription of procollagen and the COPII machinery required for ECM trafficking, notably Sec23A (Chen et al., 2014; Murakami et al., 2009; Saito et al., 2009a). Collagen synthesis in dermal fibroblasts is promoted by a similar mechanism (Ishikura-Kinoshita et al., 2012). Interestingly, it was recently shown that procollagen synthesis, including its secretory trafficking to and through the Golgi, is under circadian control, indicating another level of transcriptional regulation in matrix production (Chang et al., 2020). It is therefore clear that the ability of cells to produce ECM is sensitive to transcriptional regulation at the level of the Golgi, which can be in response to various physiological stimuli.

## The importance of Golgi function in the production of ECM – GAG modification of proteoglycans

### Synthesis of SLRP proteoglycans at the Golgi

The importance of Golgi organisation and function in producing matrix molecules, particularly with respect to glycosylation, is exemplified by the heavily glycosylated small leucine-rich repeat proteoglycans (SLRPs) biglycan (BGN) and decorin (DCN) (Chen and Birk, 2013). These proteoglycans consist of a core protein of 40 and 43 kDa, respectively, modified at the N-terminus by the addition of one (DCN) or two (BGN) chondroitin sulfate (CS) or dermatan sulfate (DS) GAG chains. They also carry N-linked glycans in their leucine-rich repeats (Chen and Birk, 2013).

The synthesis of CS or DS, as well as heparan sulfate (HS) and heparan, begins with the assembly of a common core tetrasaccharide followed by the addition of 100 or more GAG-specific repeating disaccharide units (Box 2). The sequential nature of GAG assembly is reflected in the organisation of Golgi enzymes. Chain initiation begins with the addition of xylose to specific serine residues in the core protein. Some studies report this might take place in the ER (Kearns et al., 1993; Vertel et al., 1993); however, it is likely the majority of xylose transfer occurs in the ERGIC and/or *cis*-Golgi (Jonsson et al., 2003; Kearns et al., 1993; Lohmander et al., 1989; Nuwayhid et al., 1986; Vertel et al., 1993). The core tetrasaccharide is then assembled in the *cis-medial* Golgi, while chain elongation and modification take place in the *medial-trans* Golgi (Prydz, 2015; Sugumaran et al., 1992; Sugumaran and Silbert, 1991). This organisation has been confirmed by multiple groups, showing that the machinery required for core tetrasaccharide assembly can be separated from that required for chain elongation and sulfation by using brefeldin A treatment (Calabro and Hascall, 1994; Jonsson et al., 2003; Spiro et al., 1991; Sugumaran et al., 1992; Sugumaran and Silbert, 1991; Uhlin-Hansen et al., 1997), which inhibits COPI transport and causes fusion of the early Golgi with the ER (Lippincott-Schwartz et al., 1989). Similarly, the addition of a KDEL sequence to DCN, which prevents its progression to the late Golgi, prevents chain elongation but not tetrasaccharide assembly (Jonsson et al., 2003). Sulfation is also ordered across the Golgi stack, with sulfation of the C6 and C4 positions in *N*-acetylgalactosamine occurring in the *trans*-Golgi (Hoppe et al., 1985; Sugumaran and Silbert, 1991). Meanwhile, epimerisation occurs specifically in the late *trans*-Golgi (Hoppe et al., 1985). This organisation likely ensures efficient glycan assembly and could regulate the nature or quantity of GAG chains produced.

**Box 2. GAG synthesis at the Golgi complex**

As illustrated in the figure, the initiation of chondroitin sulfate (CS), dermatan sulfate (DS), heparan and heparan sulfate (HS) synthesis begins with the addition of xylose (Xyl) to specific serine residues in the core protein. This step is catalysed by xylosyltransferase I (XylTI) (Kearns et al., 1991) and II (Cuellar et al., 2007) in the ERGIC and early Golgi (Lohmander et al., 1989; Nuwayhid et al., 1986; Vertel et al., 1993). Two galactose residues (Gal) are then sequentially added in the *cis*-*medial* Golgi by β-1,4 galactosyltransferase 7 (XGalTI) and β-1,3 galactosyltransferase 6 (GAG GalTII), encoded by the *B4GALT7* and *B3GALT6* genes, respectively. Next, glucuronic acid (GlcA) is added by β-1,3-glucuronyltransferase 3 (GlcATI, encoded by *B3GAT3*) in the *medial*-*trans* Golgi (Pedersen et al., 2000). This completes the formation of the core tetrasaccharide linkage region common to all four GAGs. Mutations in any of the enzymes responsible for building this linkage cause a set of diseases termed linkeropathies (Mizumoto et al., 2013, 2015; Paganini et al., 2019). These are multisystemic disorders of connective tissues and skeletal structures, characterised by a loss of GAG content in the ECM (see Table 1 for a summary).

The addition of a fifth sugar in the *medial*-*trans* Golgi determines which GAG is built – the addition of N-acetyl-D-galactosamine (GalNAc) by GalNAc transferase I (GalNAcTI) (Rohrmann et al., 1985; Sato et al., 2003) initiates DS or CS synthesis, whereas the addition of N-acetyl-D-glucosamine (GlcNAc) by GlcNAc transferase I initiates HS or heparan synthesis. Chain elongation then proceeds with the addition of more than 100 chain-specific repeating disaccharide units – [4GlcAβ1-3GalNAcβ1] for CS or DS and [4GlcAβ1-4GlcNAcα1] for HS or heparan. CS and DS elongation is catalysed by the enzyme complex chondroitin sulfate polymerase. This complex includes a chondroitin sulfate polymerase (ChSy), which possesses both CS-GlcATII and GalNAcTII enzymatic activities, and the chaperone chondroitin polymerase factor (ChPF). DS is then created through the conversion of GlcA to IdoA within the chain by dermatan sulfate epimerase. Heparan chains are built by the heparan sulfate polymerase, consisting of EXT1 and EXT2, and can also undergo GlcA to IdoA conversion. All chains are sulfated in the *medial-trans* Golgi concurrent with chain elongation (Sugumaran and Silbert, 1990). GAG chain synthesis and sulfation, not discussed here, is reviewed in more detail in Silbert and Sugumaran (2002). The sulfotransferases associated with disease are also summarised in Table 1.
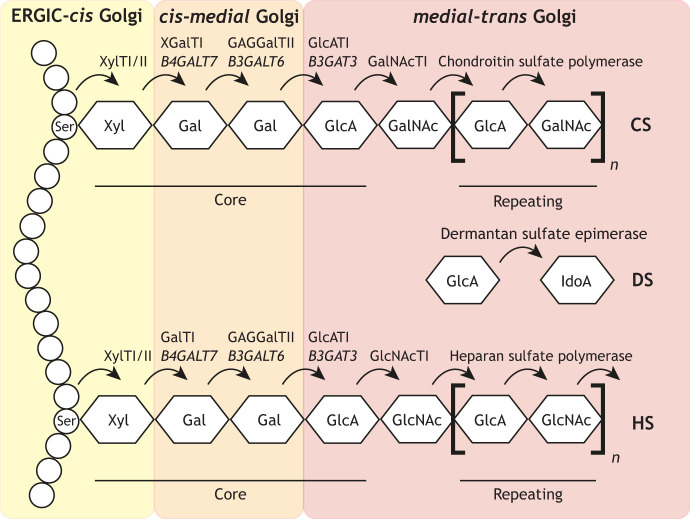

The compartmentalisation of enzymes within cisternae also supports the formation of functional enzyme complexes to facilitate efficient substrate transfer between enzymes (Dick et al., 2012). In particular, epimerases and sulfotransferases have been observed in oligomeric complexes partitioned to distinct Golgi domains. The translocation of sugars from the cytosol into the lumen of specific cisternae also acts to increase precursor concentrations 50- to 100-fold in the vicinity of the relevant glycosyltransferases (Dick et al., 2012; Hirschberg and Snider, 1987). In the case of UDP-xylose, the Golgi itself acts as a site of synthesis, reducing the reliance of GAG initiation on sugar transporters (Kearns et al., 1991; Moriarity et al., 2002). Indeed, loss of the Golgi-resident UDP-xylose synthase leads to craniofacial defects in zebrafish (Eames et al., 2010). Cisternal pH is also important in GAG production, given that neutralisation with weak bases or proton pump inhibitors increases CS and DS sulfation (Grondahl et al., 2009; Harper et al., 1986).

### The functional importance of SLRP proteoglycan synthesis

The presence, length, type and degree of sulfation of GAG chains is important for ECM and tissue function. In the case of DCN and BGN, CS is the main GAG in cartilage and bone, whereas DS is more prevalent in soft tissues, which might confer different properties relevant to the biomechanics of these tissues (Boskey et al., 1997; Yan et al., 2011). In bone, temporally regulated changes in SLRP expression during mineralisation (Takeuchi et al., 1990) and spatially regulated patterns of sulfate modification throughout the tissue (Takagi et al., 1991) all support the idea that Golgi GAG output is carefully controlled to orchestrate mineral deposition (Waddington et al., 2003). GAGs also affect the interaction of SLRPs with extracellular collagen. For example, binding to type I collagen is enhanced in the presence of GAG chains, at least *in vitro* (Schonherr et al., 1995), and DCN binds to type XII (Font et al., 1993, 1996), XIV (Ehnis et al., 1997) and VI (Nareyeck et al., 2004) collagen in a GAG-dependent way. The influence of GAG chains on fibrillogenesis, however, seems to be context specific; the presence of a DS chain on DCN can enhance (Kuc and Scott, 1997) or limit (Ruhland et al., 2007) fibril diameter *in vitro*, whereas dermatan sulfate epimerase I-null mice, which lack DS chains, have thicker fibrils in the skin (Maccarana et al., 2009).

Besides their direct influence on matrix structure, glycanated SLRPs also have important roles in signalling, which in turn is important for the differentiation and survival of matrix-producing cells. For example, BGN-mediated enhancement of bone morphogenic protein 4 (BMP-4) signalling, which is required for osteogenesis, is dependent on the presence of a GAG chain (Ye et al., 2012), and glycanation of BGN and DCN modulates availability and signalling of transforming growth factor β (TGF-β), which must be tightly controlled to maintain cell differentiation status (Hildebrand et al., 1994). A number of matrix diseases are attributable to defects in proteoglycan modification, as described further below (Table 1), underscoring the importance of this Golgi-localised process. Despite its obvious importance, we do not yet have a clear picture of how Golgi GAG chain output is controlled (reviewed in Mikami and Kitagawa, 2013).

## The importance of Golgi function in the production of ECM – transport and processing of procollagen

**Fig. 3.**
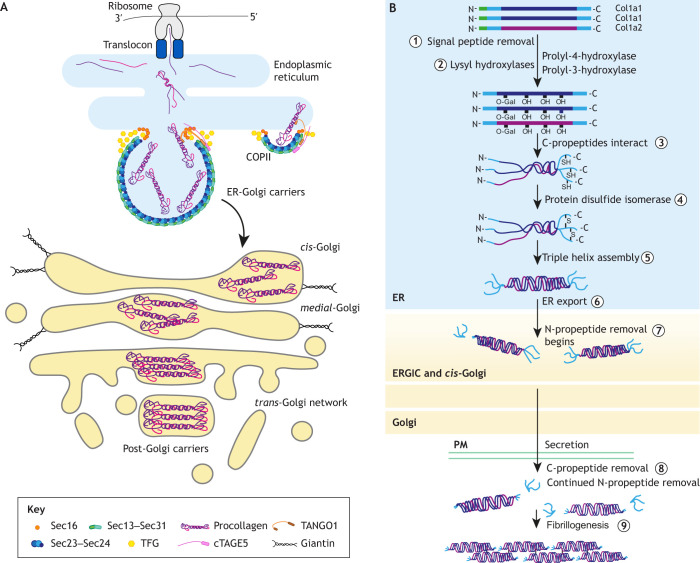
**Procollagen processing and trafficking.** (A) Schematic overview of procollagen secretion. Procollagen molecules are co-translationally imported into the ER and assemble into a triple helix (see B). They are then exported from the ER in a COPII-dependent manner. COPII assembly is driven by the core coat subunits Sec23, Sec24, Sec13 and Sec31 and is facilitated by other factors, including Sec16 and TFG. The recruitment of procollagen to the carriers is enabled by the adaptors TANGO1 and cTAGE5. Once procollagen is transferred to the Golgi, it transits through the cisternae before packaging into post-Golgi carriers for transport to the cell surface. (B) Procollagen synthesis. Type I procollagen is synthesised as two Col1a1 chains and one Col1a2 chain, which are co-translationally imported into the ER. After import, signal peptides are removed (1) and key lysine residues in the triple helical domain of each chain are hydroxylated (2). Some glycosylation also takes place. Lysyl hydroxylation initiates the folding of the trimeric procollagen molecule, beginning with the interaction of the C-terminal ends of each chain (3) and stabilisation by disulfide bond formation (4). The chains then zipper up to form a triple helical molecule flanked by globular N- and C-terminal propeptide domains (5). Immature procollagen is exported from the ER (6) and progresses through the ERGIC to the cis-Golgi. Between these compartments, N-propeptide cleavage is initiated by an unknown enzyme (7). Procollagen then progresses through the Golgi and is secreted into the extracellular space where the C-propeptide is proteolytically cleaved by mTLD, TLL1, TLL2 or BMP1 (8). N-propeptides that escape intracellular processing are also proteolytically cleaved here by ADAMTS-2, -3, -14 and/or meprin-α or -β. Following processing, the mature triple helical collagen assembles into fibrils (9).

The Golgi plays a vital role in collagen production (Fig. 3). Additionally, studying this major fibrillar matrix protein has itself provided important insight into the processes of trafficking to and through the Golgi. Following its export from the ER in COPII-coated carriers, which involves several additional factors including TFG (McCaughey et al., 2016; Witte et al., 2011), TANGO1 (also known as MIA3) (Saito et al., 2009b) and cTAGE5 (also known as MIA2) (Saito et al., 2011), procollagen is delivered, via the ERGIC, to the *cis*-Golgi (McCaughey et al., 2021). Once in the Golgi, procollagen congregates in specific dilated domains of Golgi cisternae, as shown by EM studies (Fig. 3) (Bonfanti et al., 1998; Leblond, 1989; Marchi and Leblond, 1984; Mironov et al., 2001; Trelstad and Hayashi, 1979; Trucco et al., 2004). These procollagen aggregates are only observed within cisternae and never in vesicles or intra-Golgi tubules (Trucco et al., 2004), consistent with them being too large to fit into these carriers, and instead transiting the Golgi in maturing cisternae. In certain cell types, procollagen also aligns into parallel arrays as it progresses from the *cis*- to *trans*-Golgi (Bonfanti et al., 1998; Leblond, 1989; Marchi and Leblond, 1984; Mironov et al., 2001; Trelstad and Hayashi, 1979; Trucco et al., 2004), and it maintains this organisation as it buds off into post-Golgi carriers. Concentration and alignment of the procollagen molecules within Golgi distensions thus provides a means to organise them early in their synthesis to facilitate fibrillogenesis.

Simultaneously with aggregation, procollagen acquires sugar modifications upon reaching the more *trans*-cisternae (Leblond, 1989). There is also increasing evidence to suggest that proteolytic processing of procollagen begins within the Golgi (Canty-Laird et al., 2012; Humphries et al., 2008; Stevenson et al., 2021). Fibrillar procollagen molecules consist of a central triple helical domain flanked by globular N- and C-terminal propeptide domains that are removed prior to, or concomitant with, fibril formation (Canty and Kadler, 2005; Miyahara et al., 1984) (Fig. 3). Although these processing steps can occur in the extracellular environment, pulse-chase experiments with ^14^C-labelled type I procollagen show that cleavage can start within the secretory pathway (Canty-Laird et al., 2012; Humphries et al., 2008). Consistent with this, N-propeptide cleavage can proceed in the presence of brefeldin A in tendon explants, indicating at least some N-propeptidase activity is localised to the early secretory pathway (Canty-Laird et al., 2012). Furthermore, loss of the *cis-medial-*golgin giantin (also known as GOLGB1) results in the loss of intracellular N-propeptide processing in cell culture, further implicating this compartment in this step (Stevenson et al., 2021).

The precise role of the *cis*-Golgi, and giantin, in regulating procollagen processing remains elusive; however, it is likely that this compartment is home to at least one procollagen N-propeptidase, which may require giantin for its localisation or processing. So far five N-propeptidases have been identified in mammals, which are all secreted: ADAMTS-2, -3 and -14, and meprin-α and -β (Bekhouche and Colige, 2015; Broder et al., 2013). These are all expressed as zymogens, which themselves need to be cleaved for activation. For the ADAMTS proteins, this is achieved by furin-like convertases in the TGN (Wang et al., 2003). In turn, the furin-like convertases are also expressed as inactive precursors that undergo autocatalytic activation in the TGN (Leduc et al., 1992). Interestingly, treatment of tendon explants with furin inhibitors does reduce, but does not eliminate, intracellular procollagen N-propeptide cleavage (Canty-Laird et al., 2012). Therefore, a furin-like convertase proteolytic cascade is required, at least in part, for intracellular procollagen processing, but a stable pool of active propeptidase, or an unidentified furin-independent enzyme, must also exist within the early secretory pathway to explain the experimental observations.

## Genetic disorders of the ECM linked to Golgi function

Given the importance of the Golgi in the production and maintenance of the ECM, as exemplified above, it is unsurprising that many genetic disorders caused by mutations in Golgi proteins result in matrix defects. These are typically rare, but give important insights into matrix biogenesis. In this section, we describe examples of such disorders to illustrate the different mechanisms that can underlie a matrix phenotype (Table 1, see also Fig. 2). Some of these disorders lie within the larger class of disorders known as congenital disorders of glycosylation (CDGs), which arise from defects in glycosylation and often manifest as multisystemic phenotypes (Freeze and Ng, 2011; Péanne et al., 2018; Reily et al., 2019). These have been covered elsewhere, and here, we only highlight certain examples of CDGs that result in a major ECM defect.

### Golgi enzymes

A number of disorders with a matrix phenotype can be attributed to mutations in Golgi enzymes (Table 1). For example, mutation of β-1,4-galactosyltransferase 7 (*B4GALT7*), involved in generating the tetrasaccharide linkage region of PGNs (Box 2), results in defective glycosylation of DCN and BGN (Seidler et al., 2006) in the connective tissue disorder Ehlers–Danlos syndrome, spondylodysplastic type 1 (EDSSPD1) (Faiyaz-Ul-Haque et al., 2004; Götte and Kresse, 2005; Kresse et al., 1987; Okajima et al., 1999). Collagen fibril formation is severely perturbed in EDSSPD1 fibroblasts (Seidler et al., 2006), confirming the importance of both DCN and BGN glycosylation in this process. A similar outcome was seen with mutations in the gene encoding the related enzyme β-1,3-galactosyltransferase 6 (*B3GALT6*), which causes Ehlers–Danlos syndrome, spondylodysplastic type 2 (EDSSPD2) and spondyloepimetaphysical dyspasia with joint laxity 1 (SEMDJL1) (Malfait et al., 2013; Nakajima et al., 2013; Van Damme et al., 2018; Vorster et al., 2015). Other Golgi enzymes involved in the formation of the tetrasaccharide linkage region of PGNs have also been found to be responsible for connective tissue disorders, and collectively these disorders have been termed linkeropathies (Jones et al., 2015; Mizumoto et al., 2015; Ritelli et al., 2019).

Mutation of Golgi enzymes involved in processes other than glycan processing can also result in matrix defects. An interesting example is provided by the metalloprotease S2P (Caengprasath et al., 2021), whose mutation causes the X-linked bone disorder osteogenesis imperfecta 19 (OI19) (Lindert et al., 2016). Both of the S2P mutations that have so far been identified in OI19 patients are found in intramembrane residues critical for metal ion coordination (Lindert et al., 2016). Consequently, the cleavage of its substrate transcription factor CREB3L1 is impaired. This results in reduced synthesis and secretion of procollagen, explaining the bone phenotype seen in patients (Lindert et al., 2016).

### Ion pumps and transporters

The Golgi relies on a variety of pumps and transporters to maintain correct luminal pH and ion composition, as well as to move sugars into the Golgi for glycosylation reactions. Golgi pH is crucial for the activity and localisation of glycosyltransferases (Guillard et al., 2009), and hence disruption of pH can have wide-ranging effects upon cargo modification. For example, mutation of ATP6V0A2, a subunit of the V-type H^+^ ATPase that acidifies many organelles including the Golgi (Futai et al., 2000), leads to defects in both N- and O-linked glycosylation, impaired Golgi trafficking and disruption of Golgi structure (Hucthagowder et al., 2009; Kornak et al., 2008; Udono et al., 2015). These defects manifest as autosomal recessive cutis laxa 2 (ARCL2A) (Kornak et al., 2008), characterised by wrinkly, inelastic skin and bone abnormalities, consistent with ECM changes playing a prominent role in the disease aetiology.

Disruption of Golgi ion homeostasis can result in ECM defects in other ways. For example, mutation of ATP7A, a TGN-localised ATP-driven copper ion pump, causes occipital horn syndrome (OHS), an X-linked recessive disorder that affects the connective tissue, skeleton and nervous system (Dagenais et al., 2001; Ronce et al., 1997; Tang et al., 2006). The ECM abnormalities can be explained by reduced activity of the ATP7A substrate lysyl oxidase, a cuproenzyme required for initiation of cross-linking of collagen and elastin. ATP7A mutations also cause Menkes disease, a more severe disorder that is usually fatal in childhood with connective tissue defects and progressive neurodegeneration (Møller et al., 2005; Skjørringe et al., 2017; Tümer et al., 1997). The pathological mechanisms of OHS and Menkes disease are likely conserved, with Menkes arising from a complete loss of ATP7A activity, whereas in OHS there is residual activity, resulting in a milder phenotype.

### Trafficking proteins

Mutations in Golgi trafficking machinery have been found to cause various ECM disorders (Table 1). For example, mutation of the COPI subunit β′-COP (COPB2), leading to haploinsufficiency, has recently been shown to cause a disorder characterised by osteoporosis and developmental delay (Marom et al., 2021). This is thought to be caused by disrupted procollagen trafficking to the Golgi, likely due to defective recycling of the machinery required to maintain anterograde flux of this cargo. Interestingly, mutations in β′-COP have also been attributed to the disorder microcephaly 19 (DiStasio et al., 2017). Given that recent work has shown a link between microcephaly and impaired ECM secretion (Esk et al., 2020), one might speculate that the underlying cause of microcephaly 19 could be improper trafficking of procollagen or other matrix proteins. Another disorder associated with defects in COPI traffic is the skin and bone disorder geroderma osteodysplastica (Hennies et al., 2008), caused by mutation of the COPI scaffolding protein GORAB (Witkos et al., 2019). DCN and BGN GAG chain synthesis is particularly sensitive to loss of GORAB, likely accounting for the fragility of collagen fibres in GO patients, and is also associated with altered TGF-β signalling and cell senescence within affected tissues (Chan et al., 2018).

Mutations in RIC-1, a subunit of the Golgi-localized guanine nucleotide exchange factor (GEF) for RAB6A, cause CATIFA (cleft lip, cataract, tooth abnormality, intellectual disability, facial dysmorphism, attention-deficit hyperactivity disorder) syndrome (Unlu et al., 2020). This is associated with defective procollagen secretion, accounting for the skeletal abnormalities seen in patients and RIC1-deficient animal models. Deficits in N-linked glycosylation of cartilage and neural ECM were also seen upon loss of RIC1 or its partner protein RGP1, consistent with a more widespread dysregulation of ECM production (Pusapati et al., 2012; Unlu et al., 2020).

Mutations in Golgi vesicle-tethering components can also cause disease. For example, mutation of the golgin GMAP210 (TRIP11) gives rise to two skeletal disorders in humans, namely achondrogenesis 1A (ACG1A) and odontochondrodysplasia (ODCD), which are both associated with defective trafficking and glycosylation of matrix proteins (Smits et al., 2010; Wehrle et al., 2019). ACG1A is characterised by severely deficient or absent ossification and is lethal, whereas ODCD has a milder skeletal phenotype that is accompanied by dental abnormalities. The milder phenotype of ODCD can be attributed to residual expression of GMAP210 in these patients and a milder trafficking and glycosylation defect compared to that seen with ACG1A, which is usually associated with a complete loss of GMAP210 expression (Wehrle et al., 2019). In addition to these genetic diseases, the Golgi has a key role in matrix maintenance throughout normal healthy ageing, as well as in chronic disease states such as cancer, as discussed below.

## Cancer, wound healing and age-related degeneration

The ECM is critical for maintaining tissue homeostasis and health, and dysregulation of its production and maintenance is associated with common malignancies such as cancer. The ECM is also critical for wound healing, and it is known that ECM integrity deteriorates with age. The function of the Golgi plays an important role in these major aspects of ECM biology, which is described below.

### Cancer

The ECM plays a major role in cancer (Cox, 2021). Changes in the matrix result in a number of biophysical and biochemical changes that affect cell signalling, adhesion and migration, which together impact upon tumour initiation and progression (Egeblad et al., 2010; Winkler et al., 2020). The extent to which the Golgi can impact upon matrix remodelling in cancer is unclear but given its critical role in the production of matrix its importance is implicit.

A direct link between the Golgi and cancer comes from studies of protein glycosylation, which can influence many aspects of tumorigenesis (Rodrigues et al., 2018). The GALA pathway, which is a driver of tumorigenesis, involves the relocation of N-acetylgalactosaminyltransferases (GALNTs) from the Golgi to the ER as a consequence of increased Src activity that promotes retrograde COPI traffic (Chia et al., 2014; Gill et al., 2010). The consequence is elevated production of Tn antigen, a glycoconjugate associated with tumorigenesis (Springer, 1984). Although the full mechanisms by which the GALA pathway contributes to cancer remain to be determined, a known target is the matrix metalloproteinase MMP14 (Nguyen et al., 2017), which promotes metastasis by increasing localised ECM degradation, in turn facilitating cell migration and invasion (Castro-Castro et al., 2016). This may be further enhanced by GALA-dependent export of ER oxidoreductases to the cell exterior where they act upon matrix proteins to reduce disulfide binds and increase proteolytic degradation (Ros et al., 2020).

Another way in which changes in Golgi glycosylation can result in tumorigenesis is through GOLPH3, a COPI adaptor for numerous Golgi enzymes (Eckert et al., 2014; Rizzo et al., 2021; Tu et al., 2008; Welch et al., 2021). GOLPH3 is an oncoprotein and is overexpressed in a number of cancers. GOLPH3 likely exerts its tumorigenic properties by affecting glycosylation of various proteins and lipids that influence mitogenic signalling at the plasma membrane (Sechi et al., 2020). This can alter the expression of many proteins, but one protein of interest in the context of this review is MMP9, which degrades ECM to promote cell migration and invasion, and is upregulated when GOLPH3 is highly expressed (Bera et al., 2013; Dupouy et al., 2014; Li et al., 2015).

### Wound healing and fibrosis

If tissue is damaged, upon injury for example, then wound healing takes place to repair the damage (Wight and Potter-Perigo, 2011). Wound healing relies on extensive synthesis and secretion of new matrix components and, as such, the secretory pathway plays a fundamental role in this process. However, the extent to which the secretory pathway, or the Golgi more specifically, changes during a wound healing response remains to be investigated. It is clear that the amount and composition of matrix deposited during wound healing is controlled, as excessive deposition of ECM, in particular fibrillar collagens, results in fibrosis, causing scarring and disrupting tissue homeostasis (Henderson et al., 2020). One of the symptoms associated with EDSSPD1, caused by mutation in B4GALT7, as mentioned above, is a delay in wound repair, indicating that Golgi-dependent glycosylation of DCN can influence wound healing (Seidler et al., 2006). Alongside data showing that changes in the concentration of CS and HS affect cell proliferation during wound repair (Elenius et al., 2004; Zou et al., 2004), this suggests a vital role for the Golgi in its role as a hub for post-translational modification of ECM components in the wound-healing process.

### Ageing

Ageing leads to a gradual loss of structural integrity of skin and bone as time passes (Bonté et al., 2019; Farr and Khosla, 2019). In skin, the epidermis thins and there is increased wrinkling, while bone becomes more fragile, as seen in osteoporosis. During ageing, there are changes in the relative amount of each matrix component produced, as well as the post-translational modifications these proteins receive, consequently altering matrix composition (Ewald, 2020). Many of the changes can be accounted for, at least in part, by senescence, a key signature of ageing, which is characterised by cell cycle arrest and resistance to apoptosis (Di Micco et al., 2021). Both increases and decreases in the synthesis and secretion of the key matrix constituents are seen in senescent cells, depending upon the cell type and tissue affected (Levi et al., 2020). Senescent cells have an altered Golgi structure, which might reflect changes in the functionality of this organelle (Cho et al., 2011; Despres et al., 2019). Although the mechanisms underlying such changes in Golgi organisation remain poorly understood, reduced expression of the V-ATPase subunit ATP6V0A2 mentioned above may be relevant, which could also account for the changes in glycosylation seen in senescent cells (Udono et al., 2015). As well as changes in the production of ECM proteins, senescent cells also secrete various molecules, including pro-inflammatory cytokines and MMPs as part of the senescence-associated secretory phenotype (SASP), which can further impact upon ECM integrity (Campisi, 2013; Coppé et al., 2008; Franceschi et al., 2000).

The modification and abundance of PGNs is particularly sensitive to ageing and can be used as a marker of the ageing process. Reduced glycan chain lengths are observed as tissues get older, which may reflect reduced chain stability, but equally could be indicative of reduced modification in the Golgi (Derwin et al., 2001; Grzesik et al., 2002; Lee et al., 2016; Li et al., 2013; Miguez, 2020). Similarly, total GAG abundance and expression of PGNs are reduced in aged tissues, with the former again consistent with reduced Golgi-dependent modification (Derwin et al., 2001; Fedarko et al., 1992; Lee et al., 2016; Miguez, 2020; Wang et al., 2018). Such changes can impact the matrix in several ways, for example by reducing the amount of bound water and interactions between matrix components that are required to maintain tissue strength and integrity.

## Conclusions and future directions

Clearly the Golgi plays a vital role in the production of the ECM. What is less clear is the extent to which Golgi function can be altered in response to regulatory cues, which may be intrinsic or environmental. Trafficking pathways to and from the Golgi are under circadian control (Chang et al., 2020), which is likely important for optimum production of the ECM. The Golgi also responds to its matrix-dependent mechanical environment to control lipid synthesis (Romani et al., 2019). Changes to Golgi structure and function based on the mechanical environment also impact cell migration (Pouthas et al., 2008; Thery et al., 2006; Tzima et al., 2003). These might be particularly important in wound healing, as well as in pathological situations where the tissue stiffness is typically increased, including fibrosis and cancer. Considerable data support the concept that the Golgi is not merely a permissive conduit for matrix production, but rather serves as an important control point in this process.

The control and coordination of matrix assembly remains poorly understood. Mathematical modelling of Golgi organisation and glycosylation (Fisher et al., 2019; Jaiman and Thattai, 2020; Vagne et al., 2020) presents key opportunities to understand the relative importance of different Golgi components in the process. It is also worth considering that self-correction mechanisms may exist at the Golgi, allowing for conservation of matrix homeostasis. For example, a glycan chain branching defect is corrected by the production of a structurally distinct but functionally equivalent glycan chain at the Golgi (Mkhikian et al., 2016). Similarly, loss of the golgin giantin, which is important for procollagen-I production, results in altered Golgi enzyme expression, likely as an adaptive response to altered intra-Golgi traffic (Stevenson et al., 2017).

Considering the number of diseases associated with Golgi dysfunction and aberrant matrix production, a number of potential therapeutic strategies can be considered. For monogenic disorders, gene therapy is a promising strategy (Bulaklak and Gersbach, 2020). It may be possible to treat diseases caused by defective glycosylation reactions in the Golgi using other approaches, such as dietary sugar supplementation, as recently shown for GDP-L-fucose synthase (GFUS)-CDG (Feichtinger et al., 2021). In some circumstances, local or systemic modulation of the circadian rhythm might be a useful strategy. CRISPR-based screening approaches might help us to better understand the machinery required for matrix production and modification at the Golgi, allowing the identification of new possible therapeutic targets. High-throughput screening, as recently performed in the case of procollagen-I (Calverley et al., 2020), also provides opportunities to identify new means of manipulating matrix synthesis in a therapeutic context. The wide-ranging social and economic importance of matrix biology in human health and disease makes this a key area of study with ambition to have dramatic clinical impacts.
